# Naive T lymphocytes chemotax long distance to CCL21 but not to a source of bioactive S1P

**DOI:** 10.1016/j.isci.2023.107695

**Published:** 2023-08-19

**Authors:** Nicolas Garcia-Seyda, Solene Song, Valentine Seveau de Noray, Luc David-Broglio, Christoph Matti, Marc Artinger, Florian Dupuy, Martine Biarnes-Pelicot, Marie-Pierre Valignat, Daniel F. Legler, Marc Bajénoff, Olivier Theodoly

**Affiliations:** 1 Aix Marseille University, Inserm, CNRS, Turing Center for Living Systems, LAI, Marseille, France; 2 Aix Marseille University, Inserm, CNRS, CIML, Marseille, France; 3 Biotechnology Institute Thurgau (BITg) at the University of Konstanz, Unterseestrasse 47, 8280 Kreuzlingen, Switzerland; 4 Graduate School for Cellular and Biomedical Sciences, University of Bern, 3012 Bern, Switzerland; 5 Faculty of Biology, University of Konstanz, Universitätsstraße 10, 78464 Konstanz, Germany; 6 Theodor Kocher Institute, University of Bern, Freiestrasse 1, 3012 Bern, Switzerland

**Keywords:** Immunity, Immune response, Cell biology

## Abstract

Naive T lymphocytes traffic through the organism in search for antigen, alternating between blood and secondary lymphoid organs. Lymphocyte homing to lymph nodes relies on CCL21 chemokine sensing by CCR7 receptors, while exit into efferent lymphatics relies on sphingolipid S1P sensing by S1PR1 receptors. While both molecules are claimed chemotactic, a quantitative analysis of naive T lymphocyte migration along defined gradients is missing. Here, we used a reductionist approach to study the real-time single-cell response of naive T lymphocytes to CCL21 and serum rich in bioactive S1P. Using microfluidic and micropatterning ad hoc tools, we show that CCL21 triggers stable polarization and long-range chemotaxis of cells, whereas S1P-rich serum triggers a transient polarization only and no significant displacement, potentially representing a brief transmigration step through exit portals. Our *in vitro* data thus suggest that naive T lymphocyte chemotax long distances to CCL21 but not toward a source of bioactive S1P.

## Highlights

• Adhesion of naive T cells on ICAM-1 is intermittent and not stabilized by mild flow
• CCL21 triggers stable polarity and long-range chemotaxis of naive T cells *in vitro*
• S1P-rich serum triggers a transient polarization and no significant displacement

## Graphical abstract

**Figure:**
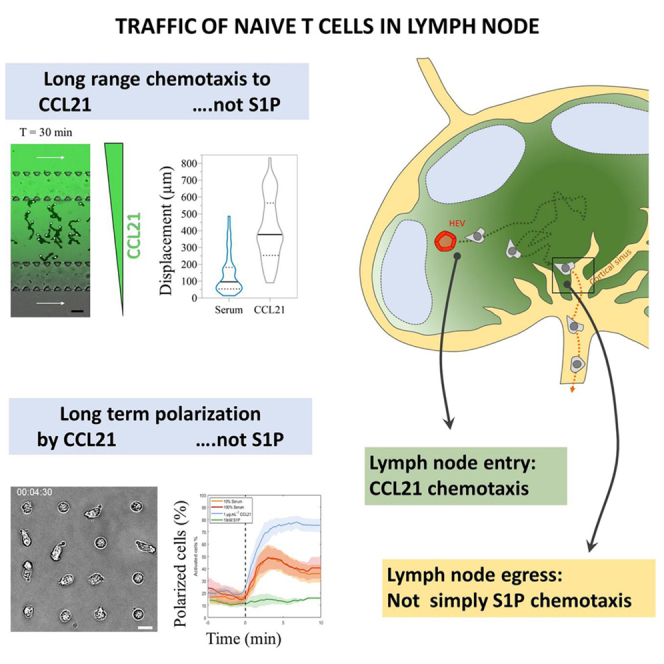

## Introduction

Chemokinesis: random migration triggered by a soluble cue.

Haptokinesis: random migration triggered by an adsorbed cue.

Chemotaxis: directed migration along a soluble cue shaped as a gradient.

Haptotaxis: directed migration along an adsorbed cue shaped as a gradient.

Naive T lymphocytes circulate through the organism in the search for cognate antigens, thereby alternating between secondary lymphoid organs (SLOs), lymphatics and the blood.^1^ Entry to and homing within SLOs are dependent on the CCR7 receptor, present on lymphocytes, and its cognate ligand CCL21 produced by stroma cells in lymphoid tissues.^1^^,^^2^ Egress from SLOs is in turn dependent on the S1PR1 receptor and its cognate ligand S1P, a small sphingolipid abundant in blood and lymph.^3^ Lymphocyte transit time through lymph nodes is thus controlled by a balance between CCL21-controlled recruitment and retention, and S1P exit signals.^4^ For this reason, disruption of the S1PR1-S1P signaling axis represents an immunosuppressing therapy applicable to a wide range of pathologies, including multiple sclerosis, transplant rejection, diabetes, and cancer.^5^^,^^6^ Surprisingly, while there is a general consensus that both CCL21 and S1P carry out their functions through chemotaxis, a quantitative analysis of naive T lymphocyte migration along defined CCL21 and S1P gradients is missing.

In the case of CCL21, lymph node gradients have been reported across organ peripheries: Across B cell follicles,^7^ along interfollicular regions,^8^ and along medullary cords.^9^ All three increase in concentration toward the central parenchyma, suggesting a single and common source: the T cell zone. Due to its positively charged C-terminal tail, CCL21 interacts with and is retained by extracellular matrix (ECM) components such as heparan sulfate or collagen.^10^^,^^11^^,^^12^^,^^13^ This capacity prevents it from being washed away during immunohistological sample preparation, allowing its visualization as a gradient, and it has been claimed that only such immobilized CCL21 triggers naive T lymphocyte migration.^14^ *In vivo,* chemotaxis of naive T lymphocytes has only been recorded along medullary cords.^9^ Other reports suggest instead random migration, at least in other regions of the lymph node.^15^^,^^16^^,^^17^^,^^18^ However, a drawback of *in vivo* experiments is that they cannot prove whether chemotaxis is triggered by a single visualized gradient or by additional yet unspecified gradients. For instance, CCL19 is another homeostatic CCR7 ligand present in lymph nodes and triggering naive T lymphocyte chemotaxis *in vitro,*^19^ but its distribution *in vivo* remains unknown because it does not bear a “sticky” tail and does not become immobilized.^20^ Since T cell zone stromal cells simultaneously produce CCL19 and CCL21,^21^^,^^22^ both chemokines are necessarily overlapping, making it difficult to judge which one is guiding naive T lymphocytes *in vivo*. Also, the fact that a chemokine may be simultaneously immobilized or soluble, coupled to the impossibility to reveal the soluble pool by staining, may further hinder the deciphering of traffic mechanisms. Such co-existences of immobilized and soluble chemokine pools have been demonstrated for the B cell zone chemokine CXCL13.^23^^,^^24^ In the case of CCL21, dendritic cells (DCs) cleave its C-terminal tail transforming it into a soluble pool that triggers chemotaxis of these cells,^25^ but has additional unique features as compared to the other CCR7 ligands.^26^ In this context, a model of chemokine cloud was tentatively proposed in which molecules are trapped as local “soluble depots” within the glycocalix.^27^ Regarding naive T lymphocytes, it remains unclear to what extent CCL19 and CCL21 guide them *in vivo*, and whether CCL21 does it as a soluble or immobilized pool.

Beyond guidance in lymph nodes, CCL21 also triggers initial extravasation of naive T lymphocytes from the blood flow into lymph nodes, through integrin-mediated adhesion to high endothelial venule (HEV) walls. It has been proposed, based on *in vitro* experiments, that external forces applied by a flow act as a critical switch triggering firm LFA-1 integrin adhesion to ICAM-1 ligands, by “inside-out” signaling.^14^ However, the interplay between flow, CCL21 and LFA-1 integrins on lymphocyte adhesion remains elusive *in vivo*, because it is unclear how a flow can exert a force on initially weakly or non-adhesive cells.

For S1P, the perspective is even more complex due to its pleiotropic functions, and since its soluble and lipidic nature impairs molecular labeling and gradient visualization. An elegant tool was recently developed in which S1P presence is deduced from the internalization rate of its receptor, which allowed for gradient identification in the spleen.^28^ However, while the same authors reported higher S1P concentrations in lymph node medullary cords than in the T cell zone, they failed to detect a gradient within or between those two areas.^29^ In addition to the lack of *in vivo* gradient identification, the percentage of naive T lymphocytes responding to S1P according to *in vitro* Transwell assays is strikingly low, typically below 10%.^4^^,^^30^^,^^31^^,^^32^^,^^33^^,^^34^ While it was claimed a consequence of its receptor’s fast internalization, this number was recently increased to almost 20% when lymphatic endothelial cells (LECs) were added to the experiments,^35^ consistent with those cells controlling a transmigration step toward S1P rather than long-distance chemotaxis toward it. Moreover, other functions have been suggested for S1P such as migration inhibition alone^36^ or migration inhibition with modulation of adhesion.^37^ Another report defends a stromal gate model where S1P acts mainly on LECs, to allow or block lymphocyte transmigration without otherwise affecting their migration.^38^ Finally, an *in vivo* report revealed that naive lymphocytes randomly approach cortical sinus exit points, with no apparent chemotaxis involved.^16^ Altogether, while chemotaxis to S1P is the prevailing model for naive T lymphocyte exit from lymph nodes, the standing evidence is conflicting.

Chemotaxis toward CCL21 and S1P remains to be faithfully demonstrated with a reductionist *in vitro* experiment where cells would migrate along a single, controlled gradient. However, the typical off-the-shelf tools in biology or immunology labs, the Transwell assays, do not properly and selectively probe chemotaxis. Transwell assays consist of two overlaid chambers, the top one containing cells and the bottom one the molecule being tested. The chambers are separated by a porous membrane through which cell transmigration is scored. While easy to handle, these assays offer no control over the gradient shape nor the moment of its arrival (time zero). They are an endpoint assay with no mechanistic insight due to the lack of *live* imaging, score transmigration through a 10–50 μm thick porous membrane without information on longer-distance gradients, and in the absence of proper controls are unable to distinguish effects of transmigration, chemokinesis, chemotaxis, or chemorepulsion.^39^ Moreover, in the case of S1P such controls are uninformative due to its receptor’s fast internalization, which precludes coincubating the molecule with the cells in the upper chamber.

The uncertainty on CCL21 and S1P guiding properties would be finally solved with *live* imaging, accessible with microfluidic tools. However, microfluidic devices for gradient generation are generally based on flow or do not control residual drifts, therefore washing away weakly or non-adhesive cells. This is the case for naive T lymphocytes, claimed to be non-adhesive on ICAM-1.^14^ To circumvent this caveat, we recently developed a microfluidic device for gradient generation in the absence of flow and used it to prove naive T lymphocyte chemotaxis toward CCL19.^19^ Here, we used high-throughput microfluidic and protein printing ad hoc approaches to dissect the response of naive T lymphocytes to immobilized and soluble CCL21 and serum rich in bioactive S1P, at the single cell level. We first show that both adsorbed and soluble CCL21 trigger naive T lymphocyte hapto- and chemo-kinesis, respectively, when presented as homogeneous chemokine fields. Next, we show that naive T lymphocytes do adhere on ICAM-1 substrates, though in a weak and intermittent way, which is not stabilized by shear stress. We finally demonstrate that CCL21 gradients trigger naïve T lymphocyte hapto- and chemo-taxis, while under similar conditions S1P-rich serum does not. Serum triggers instead a transient polarization which is consistent with a short transmigration step, rather than a long-distance attraction.

Importantly, there is a long-standing call for better understanding the human immunology.^40^^,^^41^ With most of the aforementioned evidence arising from mouse models and a recent report indicating differences between both species,^42^ plus the numerous therapeutic opportunities expected from a better understanding of the S1PR1-S1P signaling axis, we carry our studies on naive T lymphocytes purified from healthy human donors.

## Results

### CCL21 triggers naive T lymphocyte hapto- and chemo-kinesis

**Figure 1:**
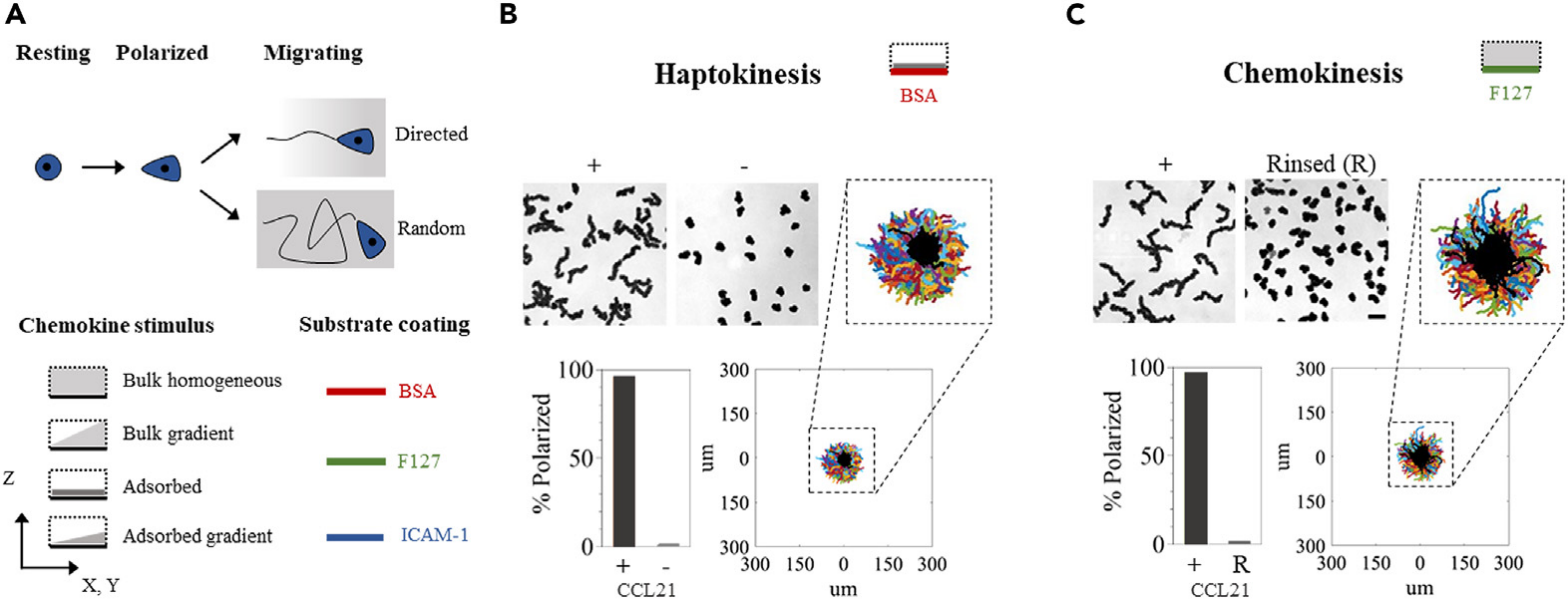
CCL21 triggers hapto- and chemo-kinesis of naive T lymphocytes (A) Top, scheme representing the process of cell migration upon stimulus sensing, from an unpolarized state (Resting) to cell polarization through symmetry breaking (Polarized), and final cell displacement in either a directed or random trajectory (Migrating). Below, cartoon description as used in all subsequent figures, describing stimulus distribution nature and substrate coating color-code. The dotted frames represent the limits of the experimental chamber, from a transversal viewing point. (B and C) Top row, bright field 10-min time projections of cells in the presence (+) or absence (−) of CCL21 adsorbed on BSA (B, haptokinesis) or kept in bulk solution over an antifouling F-127 substrate (C, chemokinesis). Bottom, Quantification of cell polarization in each condition and trajectories aligned in the origin for cells tracked over a 16-min period, in the presence (colored lines, > 500 tracks are shown) or absence (overlaid in black, > 500 tracks are shown) of chemokine. For the last condition, bulk CCL21 was rinsed (R) and fresh cells were added to detect potentially adsorbed chemokine. Data are from one representative donor, nBSA = 2, nF127 = 3 donors. Scale bar represents 30μm.

Based on *in vitro live* imaging, it has been claimed that CCL21 does not stimulate naive T lymphocytes unless adsorbed on a substrate.^14^ Since our microfluidic device creates soluble gradients based on diffusion, we first tested whether CCL21 triggered migration of human naive CD4^+^ T lymphocytes while in bulk solution only. Upon chemokine sensing, rounded naive T lymphocytes may break symmetry (cell polarization) and displace (cell migration) in a directed or random fashion according to the stimulus detected (Figure 1A), while unresponsive cells develop no motion and display pure Brownian diffusion.^43^ Cell polarization is therefore a first indicator of cell response, while accessing finer migratory parameters requires further tracking and analysis. We first analyzed the behavior of cells in non-adherent single channels without ICAM-1 or other adhesion ligands on the surface. Channels were either coated with the chemokine, rinsed and blocked with BSA (Figure 1B, adsorbed CCL21), or coated with pluronic F127, which keeps the chemokine and cells in solution^43^ (Figure 1C, bulk CCL21). We observed cell polarization and random migration in both conditions, demonstrating the molecule’s hapto- and chemo-kinetic potential (Figure 1 and Video S1). To verify that CCL21 had not permanently adsorbed (immobilized) on the F127 substrate, channels were rinsed at the end of the experiment and fresh cells were added. We observed only 2% migrating cells, proving that the chemokine had been rinsed and thus suggesting that the observed effect was triggered from molecules in the bulk solution. Based on these results, we conclude that CCL21 triggers efficient cell migration when presented in solution, and therefore can be used in our microfluidic device for studying naive T lymphocyte chemotaxis. In addition, migration in the absence of adhesion reveals the swimming capacity of naive T lymphocytes, as previously reported for effector T lymphocytes, ameba and neutrophils.^43^^,^^44^^,^^45^

Video S1. Adsorbed and bulk CCL21 trigger Naive T lymphocyte random migration, related to Figure 1

### Naive T lymphocytes intermittently adhere on ICAM-1

**Figure 2:**
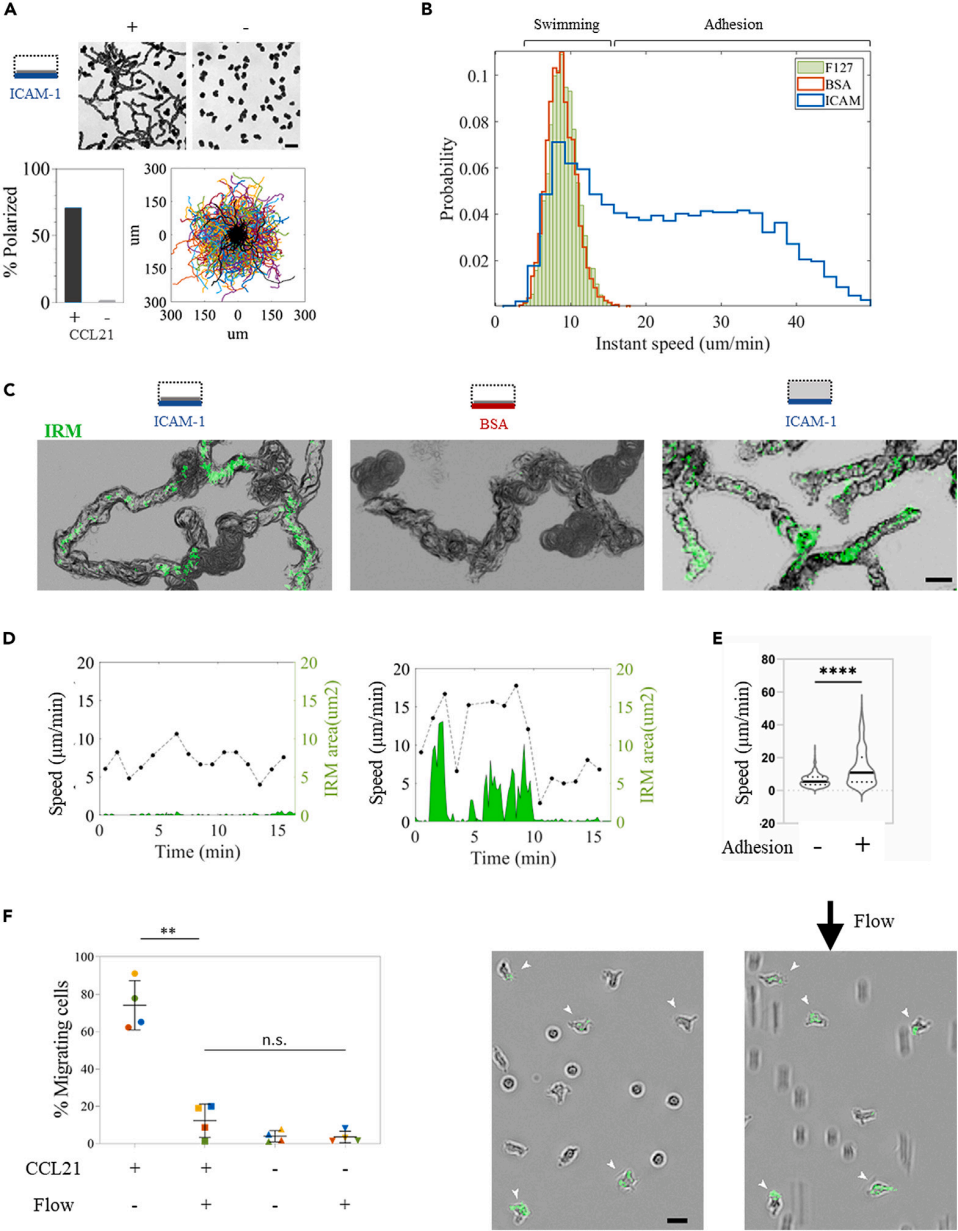
Naive T lymphocytes intermittently adhere to immobilized ICAM-1 (A) Top row, bright field 5-min time projection of cells in the presence (+) or absence (−) of CCL21 adsorbed on ICAM-1. Scale bar represents 30μm. Bottom, Quantification of cell polarization in each condition plus trajectories aligned in the origin for cells tracked over a 16-min period, in the presence (colored lines, >1000 tracks) or absence (overlaid in black, >1000 tracks) of chemokine. Data are from the same donor as in Figure 1, n > 3 donors tested. (B) Instantaneous speed distribution calculated over 1-min intervals, for migrating cells in Figures 1 and 2A n = 11709, 9256, and 5095 values calculated for ICAM-1, BSA, and F127, respectively. (C) Overlaid bright field (gray) and inverted IRM (green = adhesion patch) time projections, to reveal adhesion fingerprints while migrating under the indicated conditions. n > 3 donors tested. Scale bar represents 10μm. (D) Single cell analysis of instantaneous speed (over 1-minute intervals) and adhesion area over time, for one representative cell on BSA (Left) and ICAM-1 (Right) substrates. (E) Single cell instantaneous speed (over 10-s intervals) with and without adhesion (as detected by IRM), for cells that are polarized and have an average speed (over 200s) larger than 10 μm/min. Data from 1 representative experiment, ncells > 50, nevents> 700. ∗∗∗∗ indicates two-sided p value < 0.0001 with the nonparametric unpaired *t*-test. All data are mean + SD. (F) Left side, percent of migrating cells remaining on ICAM-1 substrates with or without adsorbed CCL21 after addition of 0.2dyn flow. Each color represents an independently tested donor. ∗∗ indicates a p value <0.01 in a multiple comparison ANOVA test. Right side, overlaid bright field (gray) and inverted IRM (green = adhesion patch) snapshots the instant before and 20 s after flow initiation. Arrowheads point at adherent cells present before and remaining after flow initiation, non-adherent cells are washed away by flow. Scale bar represents 10μm.

It has also been claimed that naive lymphocytes do not adhere on ICAM-1 in the absence of shear stress.^14^ However, when we imaged cells in single channels coated with ICAM-1 and CCL21, we noted that they explored a greater surface area (Figure 2A and Video S2). Instantaneous speed analysis revealed two populations on ICAM-1 substrates (Figure 2B), which suggests that cells with low speed may lack adhesion and swim,^43^ whereas cells with corresponding to ones adhering on the ICAM-, as reported for effector cells.^46^ We therefore performed interference reflection microscopy (IRM) to characterize such populations. With this imaging technique, cells in close contact to the substrate display destructive optical interference leading to an intensity darker than the background, whereas non-adherent cells present constructive optical interference leading to a brighter intensity. While cells migrating on BSA barely presented adhesion fingerprints, cells on ICAM-1 sequentially alternated between adherent and non-adherent states (Figure 2C). Intermittent adhesion did not arise from a shortage of adsorbed chemokine since similar results were obtained when the chemokine was kept in bulk solution, at 1 μg/mL concentration, over the ICAM-1 substrate (Figure 2C and Video S3). Single cell analysis of instantaneous speed and adhesion area (Figures 2D and 2E) confirmed that fast migration events concerned polarized cells that are adherent and crawling (14.2 ± sem 0.6 μm/min), whereas non-adherent cells had a slower speed is consistent with a swimming mode (6.2 ± sem 0.2 μm/min).^43^ Finally, application of a 0.2 dyn shear stress did not stabilize adhesion^14^ but rather washed cells away when switching into the swimming regime (Figure 2E and Videos S4 and S5). Based on these results, we conclude that naive T lymphocytes do adhere on ICAM-1, though in an intermittent fashion which is not stabilized by shear stress or chemokine availability.

Video S2. Naive T cells explore greater distances and migrate faster on ICAM-1 substrates, related to Figure 2A

Video S3. IRM imaging attests Naive T lymphocytes adhere intermittently on ICAM-1 substrates, even in the presence of CCL21 excess (1 μg/mL) in bulk solution, related to Figure 2C

Video S4. Shear stress does not stabilize Naive T lymphocyte intermittent adhesion, related to Figure 2E Low magnification bright-field imaging.

Video S5. Shear stress does not stabilize Naive T lymphocyte intermittent adhesion, related to Figure 2E High magnification bright-field and IRM imaging.

### CCL21 gradients trigger naive T lymphocyte hapto- and chemo-taxis

**Figure 3:**
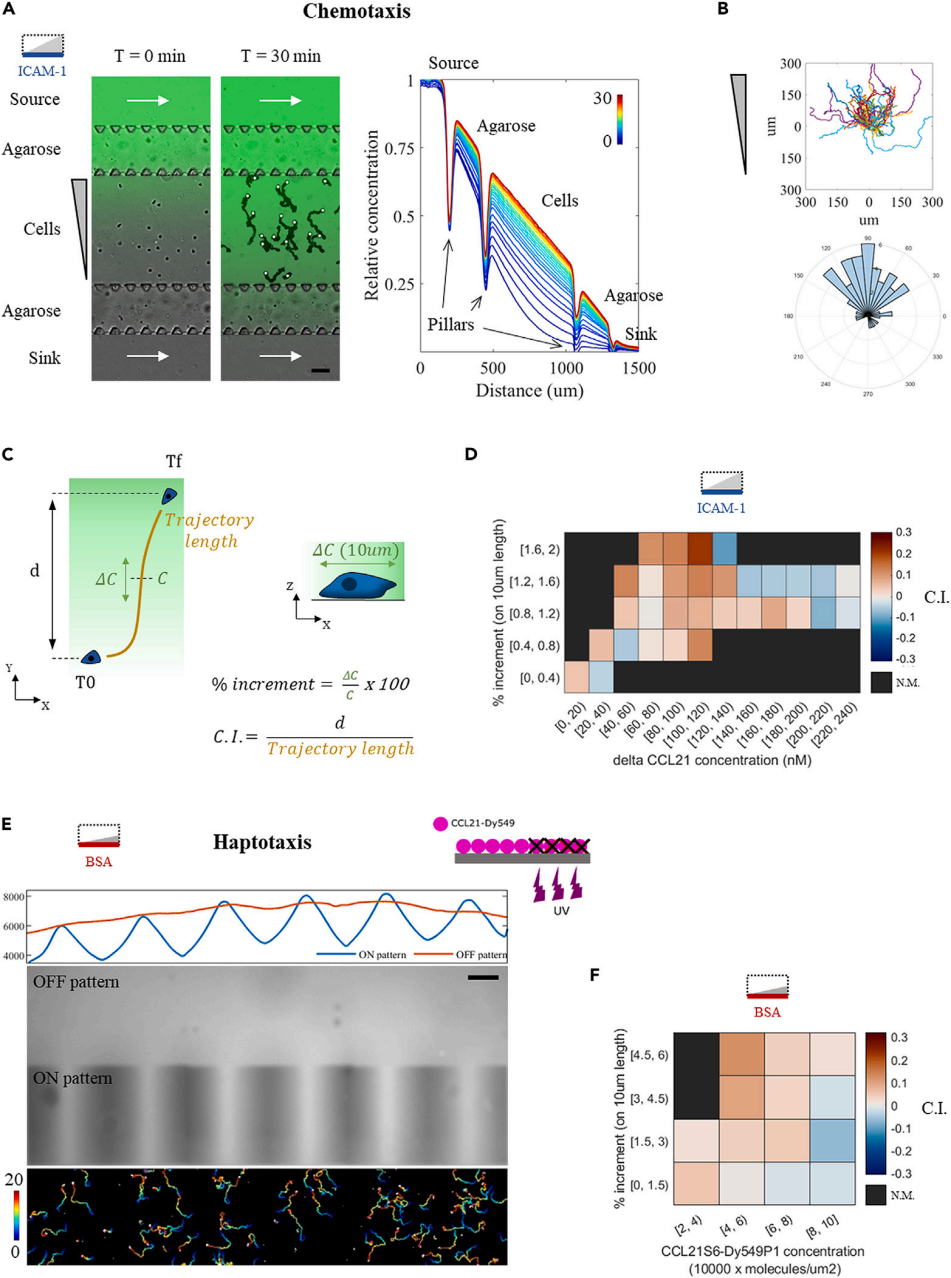
CCL21 gradients trigger hapto- and chemo-taxis of naive T lymphocytes (A) Left, overlaid bright field and fluorescent signal for an illustrative CCL21 gradient at time zero and after 30 min of acquisition. For the later, bright field images were projected to highlight cell trajectories, the white circles indicating their final position. White arrows indicate flow direction in the side channels, replenishing the source and clearing the sink to keep them at maximum and minimum chemokine concentrations, respectively. Agarose barriers, held by trapezoidal PDMS pillars, allow chemokine diffusion while hampering fluid flow across the central channel. Fluorescent 10 Kda Dextran is used to verify gradient establishment and lack of flow. Scale bar represents 100μm. Right, normalized gradient profiles taken at 1-min intervals, the color-code representing time from 0 to 30 min. Opaque PDMS pillars appear as fluorescent signal drops. Cells experience increasing concentrations of chemokine, ranging from 0 to 75% of the concentration at the source. (B) Trajectories aligned in the origin and angle histogram for a total of 55 cells from one representative donor, tracked over 50 min. N = 4 donors tested. (C) Cartoon illustrating the calculation of the chemotactic index (C. I.) as a ratio between the net displacement along the gradient direction (d) and the trajectory length. Each value is then tagged with the local chemokine concentration (C) and slope (ΔC) over 10μm length, the typical body size for a naive T lymphocyte. (D) Heatmap for the chemotactic Index (C.I.) as a function of ΔCCL21 bulk concentration and gradient slope for 3 independently tested donors (n = 215 tracks, 6422 C.I. values calculated over 1-min intervals). N.M. = not measured or below a threshold of 25 values. (E) Top, cartoon illustrating the subtractive printing protocol. Below, fluorescent image and profiles along the patterned area (ON pattern) or out of it (OFF pattern). Bottom, cell trajectories on the patterned area, color-coded with time over a 20-min period. (F) Heatmap for the chemotactic Index (C.I.) as a function of chemokine substrate concentration and gradient slope for 3 independently tested donors (n = 6809 tracks, 7878 C.I. values calculated over 1-min intervals). N.M. = not measured or below a threshold of 25 values.

Given that bulk CCL21 triggered naive T lymphocyte chemokinesis, we next tested whether it also triggered their chemotaxis by using our microfluidic device for soluble gradient generation.^19^ In our setup, cells are injected in a central channel and their behavior is recorded in response to a gradient established by diffusion (Figure 3A). Parallel channels on each side are used as the chemoattractant source and sink; they are separated from the central one by a double array of trapezoidal pillars holding permeable agarose barriers, which allow chemokine diffusion while dampening flow across them. A mild flow assures chemokine replenishment and removal at the source and sink channels, respectively, while the width of the central channel imposes the gradient steepness, with profiles spanning 500 to 1000 μm in length.^19^ When CCL21 was applied as a soluble gradient, we observed marked directional migration toward increasing chemokine concentrations (Figure 3B and Video S6). However, because the chemokine adsorbs on various substrates (Figures 1 and 2), this effect could arise from a combination of the applied soluble gradient plus a potential haptotactic one building up during the experiment, due to molecules instantly captured on the substrate. We thus turned into self-made chemokine versions^47^ to identify each contribution.

Video S6. CCL21 gradients trigger Naive T lymphocyte chemotaxis, related to Figure 3B (Green) fluorescent 10 Kda Dextran is used as a tracer to verify gradient establishment and lack of flow.

To determine the soluble contribution, we used a truncated variant, CCL21^24−102^ or ΔCCL21, lacking the C-terminal basic motif and therefore expected to remain exclusively in bulk solution. Because its diffusion is represented by a similar weight FITC-Dextran tracer, fine analysis of Chemotactic Index (C.I.) versus gradient concentration and slope was achieved (Figure 3C). Chemotaxis was maximum at high slopes and 80–120 nM concentrations, but detectable from a 0.4% increment over a cell body-length (Figure 3D). To determine the haptotactic contribution, we then used a fluorescently labeled CCL21, CCL21-S6^Dy549P1^. Starting from a homogeneously adsorbed chemokine substrate as in Figure 1A, we used a subtractive printing protocol^48^^,^^49^ to degrade chemokine functionality and create patterns of interest in various slopes and intensity ranges (Figure 3E). Since leukocyte migration is biased by gradients of adhesion ligands,^48^ we performed these experiments in the absence of adhesion. The fluorescent signal was then transformed into number of adsorbed molecules with the aid of a calibration curve (Figure S1). When correlated to the calculated C.I. values, highest haptotaxis was identified at highest slopes and concentrations of 4–6 x 10^4^ molecules/μm^2^ (Figure 3F). Based on these results, we conclude that CCL21 effectively triggers naive T lymphocyte hapto- and chemo-taxis.

### Serum modulates S1PR1 surface expression on naive T lymphocytes

**Figure 4:**
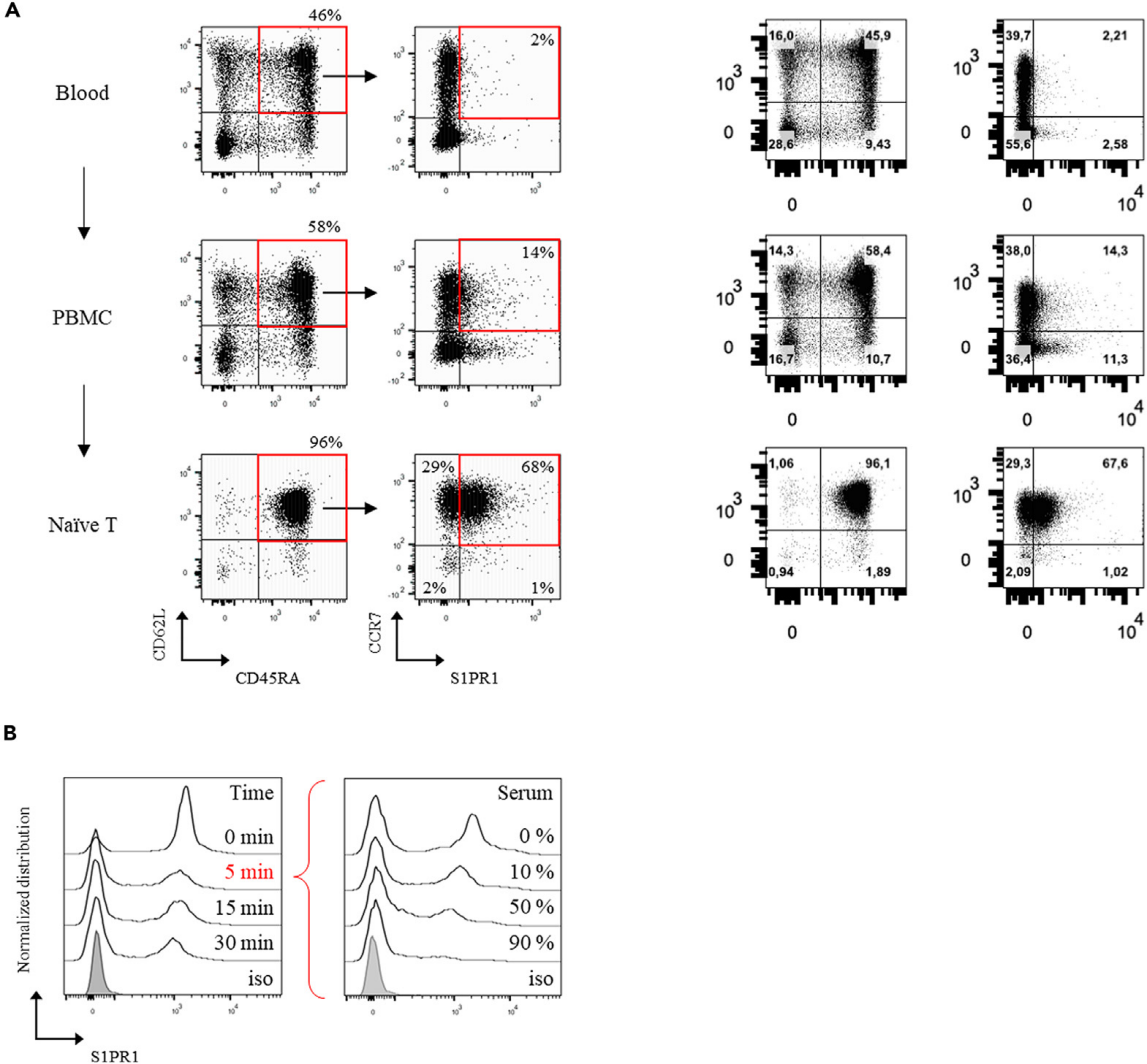
Serum modulates S1PR1 surface expression on naïve T lymphocytes (A) S1PR1 live staining performed on whole blood cells, PBMC’s and naive T lymphocytes, along the purification process. On the left panels, red squares and arrows indicate the gating strategy on CD62L + CD45RA + cells, with the population percentage indicated on top of each quadrant and increasing along the purification process. On the right panels, red squares indicate the percentage of resensitized (S1PR1+) naive T lymphocytes (CCR7+), also increasing along the purification process. (B) Left, S1PR1 modulation on cells exposed to 50% serum concentration, fixed at the indicated time points and stained. Right, S1PR1 modulation on cells exposed 5 min to the indicated serum concentrations then fixed and stained. Gray histograms correspond to isotype controls. Data are representative from 1 out of 3 and 2 independently tested donors, respectively.

Naive T lymphocytes are assumed to exit lymph nodes following a gradient of S1P. However, directed migration toward S1P was never imaged, neither *in vivo* nor *in vitro*. We therefore decided to challenge this idea using our microfluidic device. Due to its lipidic nature, S1P is carried in blood by albumin and apolipoprotein M, each of them having apparent distinct functions and cellular targets.^50^^,^^51^ Those carriers are not yet elucidated for lymph, thus it is not known in which state naive T lymphocytes encounter and sense S1P at cortical sinus exit points, which might explain the low transmigration values reported in the literature. Following this consideration, and since lymph is mixed with blood at the thoracic duct and both fluids trigger similar S1PR1 internalization,^52^ we tested fresh autologous donor serum as the source of bioactive S1P. In this way, we also sought to reduce inter-donor variability due to the use of human cells. Because experiments in mice proved that S1PR1 internalizes within minutes of exposure,^30^ we first sought to characterize its dynamics in human cells. In agreement with the literature,^53^ naive T lymphocytes did not express S1PR1 when directly stained in blood, but re-exposure occurred during cell purification (Figure 4A). When incubated in the absence of fetal calf or human serum, cells remained viable (Figure S2A) and S1PR1 exposure reached a plateau with 84 ± 8% resensitized cells (Figure S2B). When resensitized cells were then exposed to autologous human serum, we observed a fast, concentration dependent S1PR1 internalization within 5 min (Figure 4B). These results thus validated serum as a source of bioactive S1P, which was used for subsequent experiments, and serum-starved naive T lymphocytes as a relevant model for S1PR1 expressing cells.

### Serum gradients trigger transient polarization and chemokinesis of a fraction of cells, but not chemotaxis

**Figure 5:**
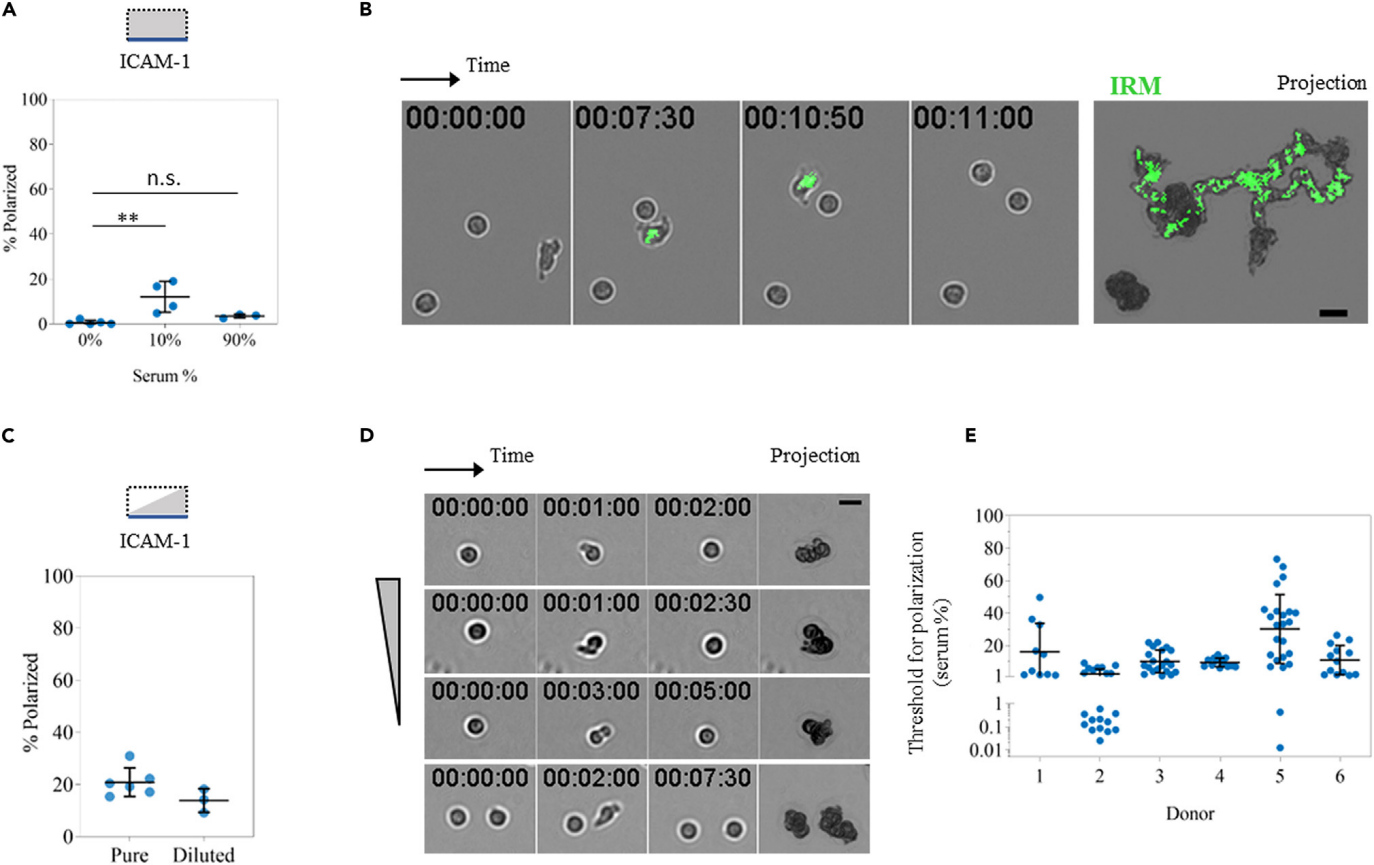
Serum gradients trigger transient polarization and chemokinesis of a fraction of cells, but not chemotaxis (A) Cell polarization in single channels in the presence of the indicated serum concentrations. A total of 664 cells from 5 independently tested donors were imaged. ∗∗ indicate a p value < 0.01 measured by multiple comparison ANOVA test. All data are mean + SD. (B) Time sequence and overlaid projections for bright field and inverted IRM imaging (green = adhesion patch) for a group of resensitized cells in 10% serum. (C) Quantification of cell polarization upon serum gradient arrival. 620 cells from 6 independently tested donors were exposed to pure serum (0–70% concentration range), while 395 cells from 3 independently tested donors were exposed to diluted serum (0–15% concentration range). All data are mean + SD. (D) Time sequence and projections of 4 representative cells shortly polarizing upon serum arrival, but without net displacement. Scale bars represents 10μm. (E) Threshold for cell polarization, defined as the serum concentration at the instant of symmetry breaking, for the 6 donors exposed to undiluted serum gradients. Values below 1% are plotted in a log scale. Each point in the dot blots represents one independently tested donor.

We first analyzed the response of resensitized cells to serum in single channels coated with ICAM-1. We observed a mild but significant effect for 10% serum concentration, with 12 ± 7% polarizing cells (Figure 5A). IRM imaging proved migrating cells were intermittently adhering, ruling out the hypothesis of adhesion inhibition (Figure 5B and Video S7). In addition, when cells were simultaneously exposed to both S1P-containing serum and CCL21, no apparent inhibition of cell migration nor adhesion was observed. In line with a faster receptor internalization at high serum concentrations (Figure 4B), we observed only 3.5 ± 1% polarized cells with 90% serum concentration. Because many of the responding cells were already polarized when starting the acquisition, we assume the response occurred and was completed during cell sedimentation, typically few minutes long. This result highlights the importance of controlling and visualizing the instant of stimulus addition (time-zero), achievable only in controlled microfluidic setups.

Video S7. 10% serum triggers Naive T lymphocyte transient chemokinesis and intermittent adhesion, but only on a small fraction of cells, related to Figure 5B

We then exposed resensitized cells to controlled serum gradients in our microfluidic device. By visualizing the moment of serum arrival, we observed a slightly stronger effect than in single channels, with 21 ± 6% cells polarizing upon serum arrival (Figure 5C). For many of them though the effect lasted few minutes, shortly polarizing on the spot without a net displacement (Figure 5D and Video S8). Indeed, only 48 out of 620 imaged cells (7.7%) displaced more than 20 μm (2 body lengths) and were tracked. Track duration was short, with a median of 11 min, therefore limiting cell displacement to 100μm (Figure S3). As a comparison, CCL21 tracks ended either when the cells reached the channel’s upper limits or when the movie, typically 50 min long, was over. Migrating cells did not exhibit a marked directionality toward the serum source, albeit the distribution of C.I. values was slightly skewed toward it. Because our gradients are built by gradual arrival of diffusing compounds, a threshold for polarization was determined by measuring serum concentration at the time of symmetry breaking. We observed a strong variation between and within individual donors, however, many cells proved sensitive to less than 10% serum concentration (Figure 5E). Since receptor saturation at high concentrations lowers the C.I. (as exemplified with ΔCCL21 in Figure 3D) and in the case of serum causes faster internalization (Figure 4B) with an expected lower number of responding cells, we finally diluted the serum in culture medium and exposed cells to unsaturating gradients. The observed effect though was weaker, with only 14 ± 5% polarizing cells (Figure 5C). Altogether, we conclude that under the same experimental conditions in which CCL21 triggers long-range chemotaxis of most cells, serum rich in bioactive S1P does not. Instead, serum is shortly chemokinetic on a small fraction of cells, while the transient polarization and lack of displacement of the remaining fraction of cells rather points toward a decision-making function.

Video S8. Serum gradients trigger transient polarization of a small fraction of cells, but not chemotaxis, related to Figure 5D Concatenated image sequences of 10 responding cells, organized by the duration of the polarization event. The yellow triangle represents the direction of the gradient.

### Instant exposure to serum transiently polarizes naive T lymphocytes

**Figure 6:**
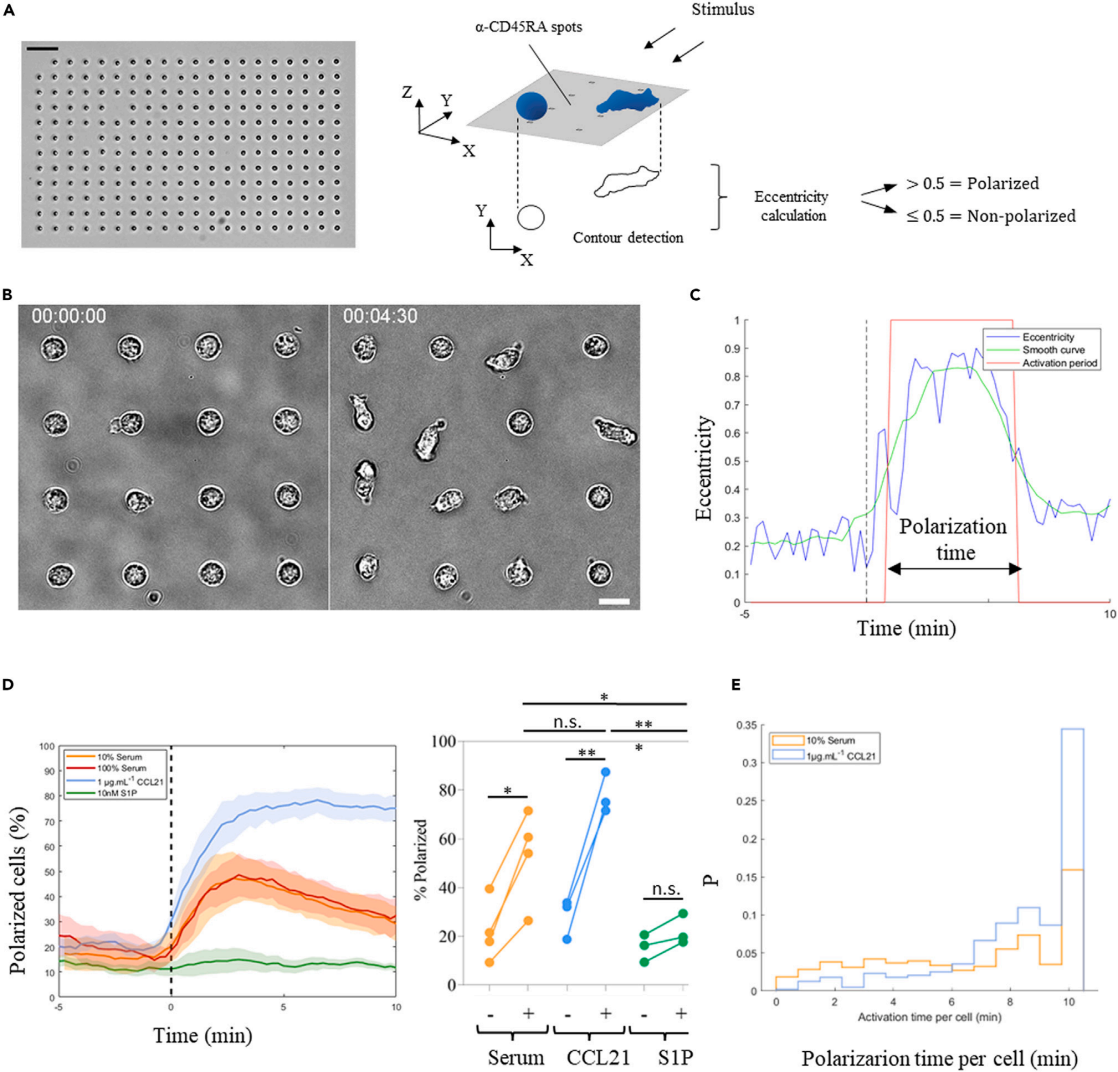
Instant exposure to serum transiently polarizes naïve T lymphocytes (A) Left side, low magnification bright-field image of a substrate after cell capture. Scale bar represents 50 μm. Right side, cartoon exemplifying the experimental setup and the morphometric analysis used to score individual cell polarization states. Cells were considered polarized when their eccentricity was higher than 0.5. (B) High magnification bright-field images of 16 representative cells from one donor at Time-zero and 4.5 min after stimulation with 10% serum. Scale bar represents 10 μm. (C) Example of shape analysis versus time for one representative cell, with instant cell eccentricity in each frame (blue), smoothed cell eccentricity averaged over 3 sequential images (green), and cell polarization state defined by an eccentricity threshold of 0.5 (red) and used to calculate its polarization time. (D) Left side, quantification of cell polarization versus time for the indicated stimulus. The first 5 min (before t = 0 min) serve as control without stimulation. Data are from pooled independent experiments; lines correspond to their average and shaded areas to standard deviations. Because no difference is observed between the two serum concentrations, only 10% serum is shown in subsequent plots. Right side, quantification of cells that have polarized during the 5 min before (−) and after (+) injection of each stimulus. n.s. = not significant; ∗, ∗∗, and ∗∗∗ indicate p values lower than 0.05, 0.01, and 0.001 measured by multiple comparison ANOVA test. (E) Histograms of polarization time per cell during the 10 min of stimulation for 10% serum and CCL21. S1P was not included in the analysis due to a lack of events. Each condition was tested on at least 3 donors, n = 447, 652, 440, and 506 imaged cells for CCL21, 10% serum, 100% serum, and S1P, respectively.

Gradient experiments attained a maximum of only 21% responding cells (Figure 5C), as opposed to the global S1PR1 internalization observed in flow cytometry (Figure 4B). Since in our device serum is gradually arriving by diffusion, we hypothesized that non-reacting cells may internalize S1PR1 before reaching the threshold for a response. We thus sought to expose them to serum in an instant manner while recording their behavior. We captured cells on the substrate to prevent their flushing upon stimulus addition, by generating an array of 240 circular capture spots coated with α-CD45RA antibodies, hereby increasing the experimental throughput while keeping a single-cell analysis, and performed quantitative morphometry of cell contours to detect changes in their polarization state over time (Figure 6A). Under such conditions, when cells were instantly exposed to serum we indeed observed fast polarization of cells (Figure 6B and Video S9). Quantification of cell polarization revealed no difference between 10 and 100% serum concentrations, which triggered polarization of an astonishing 53 ± 19% of the total cell population, and no statistical difference with CCL21 which triggered polarization of 78 ± 8% of the total population (Figure 6C). Moreover, we observed no significant effect with a 10nM commercial S1P solution, confirming that either it requires a carrier molecule or that additional factors are present in serum triggering cell polarization (Figure 6C). Finally, polarization with serum was shorter than with CCL21, as revealed by a fraction of cells with polarization times smaller than 5 min (Figure 6D). Altogether, these data confirm that serum is an efficient source of bioactive factors and suggest that (i) serum polarizes cells more efficiently when sensed in an instant fashion than as a slowly diffusing gradient, (ii) serum triggers a more transient response as opposed to the one triggered by CCL21, and (iii) commercial S1P alone is not bioactive. We therefore conclude that serum factors, whether S1P coupled to a carrier molecule and/or others, provide a transient and qualitative decision-making signal which is substantially different from the durable and quantitative guiding signal triggered by CCL21.

Video S9. Instant serum arrival transiently polarizes Naive T lymphocytes, related to Figure 6B Image sequence of 16 representative cells captured on the substrate and exposed to 10% serum.

## Discussion

Naive T lymphocyte recirculation through lymph nodes is a critical feature of the immune system at steady state condition. It is required for the vast repertoire of cells to screen for a cognate antigen-loaded DC, and it represents a crucial therapeutic target for various pathologies. CCL21 and S1P orchestrate this continuous trafficking, as deduced from perturbation experiments and *in vivo* imaging, and have been claimed chemotactic. However, to our knowledge no migration assay along a single and controlled gradient, coupled to *live* imaging, has been reported so far for naive T lymphocytes. Here, we used customized *in vitro* setups to perform *live* imaging of human naive T lymphocytes migrating under defined environmental conditions. By exposing cells to controlled and single stimuli, we provide a quantitative directionality analysis for migrating naive T lymphocytes that shed new light in the field of lymphocyte migration.

We first demonstrated that, contrary to the literature,^14^ CCL21 signals on naive T lymphocytes not only when adsorbed but also in bulk solution. Because chemokine adsorption is based on electrostatic interactions, our assays do not exactly discriminate whether those molecules are truly read from the substrate or slowly desorbing into a local soluble pool, in which case the substrate would only act as a chemokine depot. Future assays with a molecule faithfully bound to the substrate, such as through a biotinylated linker, should help answering this question. Regardless the mechanism under play, we verified the widely accepted haptokinesis and haptotaxis of naive T lymphocytes in response to adsorbed CCL21, and we recorded their chemokinesis and chemotaxis in response to bulk CCL21, providing fine directionality analysis for both cases of taxis. Chemotactic indexes were higher for soluble gradients than adsorbed ones in the conditions tested in this work. More generally, cell directivity proved in a similar range as that previously measured in response to CCL19,^19^ but lower than that reported for DCs migrating toward both CCR7 ligands.^54^^,^^55^ Such values may reflect the importance of CCR7 guidance for antigen-loaded DCs, for which a ballistic motion is needed to efficiently reach lymph nodes, whereas naive T lymphocytes are gently attracted to areas of antigen presentation while keeping a screening behavior.

Our data also dismiss the reported lack of naive T lymphocyte adhesion on ICAM-1.^14^ By using IRM imaging, we proved that the speed of naive T lymphocytes is modulated by intermittent adhesion on ICAM-1. This is in line with *in vivo* data showing that LFA-1 is necessary to sustain fast migration in lymph nodes.^34^^,^^56^ Interestingly, speed fluctuations were reported for migrating T lymphocytes in mouse lymph nodes,^15^ and migration arrests were further correlated to intracellular calcium peaks *in vivo* and *in vitro*, at least for effector T lymphocytes in confined environments.^57^ Given that human neutrophils also modulate their adhesion while migrating on ICAM-1 substrates,^44^ it is tempting to speculate whether both cell types bear an internal clock controlling such adhesion runs, hence representing an intrinsic and intended feature. In the case of naive T lymphocytes, this feature may prevent excessive adhesion on ICAM-1-expressing DCs and ensure antigen screening continuation. Finally, in our experiments intermittent adhesion was not stabilized by mild flow, in contrast to previous conclusions from similar *in vitro* experimentation,^14^ nor by an excess of bulk chemokine. An appealing hypothesis is that stable adhesion under flow may be achieved by adding a selectin-mediated rolling step, in line with the long-established adhesion cascade for leukocyte transmigration through HEVs.^58^

Finally, we analyzed the real-time response of naive T lymphocytes to a source of bioactive S1P. After almost two decades of research, S1P biology remains a controversial topic with several standing models explaining lymphocyte exit from lymph nodes. Models claiming chemotaxis toward S1P are based on Transwell assays showing weak *in vitro* transmigration values, likely due to either premature exposure to suboptimal S1P concentrations and concomitant receptor internalization, or lack of an appropriate carrier molecule for commercial S1P. Here, by visualizing cells’ behavior in real-time, we observed the vast majority of cells not responding to gradients of bioactive S1P, a small fraction transiently polarizing on the spot but without displacement, and a smaller fraction migrating with only a mild skew in directionality toward the gradient source. Interestingly, because our gradients are built by gradual diffusion and likely also causing premature S1PR1 internalization, these behaviors matched the reported values from Transwell assays: the observed 8% migrating cells recapitulate the transmigration values reported toward S1P alone,^4^^,^^30^^,^^31^^,^^32^^,^^33^^,^^34^ while the total 21% polarizing cells recapitulate the transmigration values reported toward S1P together with LECs.^35^ This correspondence suggests that cells polarizing in the spot may represent a commitment to transmigrate through (and most likely aided by) LECs, through optimized S1P-sensing conditions. Indeed, when cells were instantly exposed to serum the percent of polarizing cells increased to ∼50%, to our knowledge the highest effect reported to date. Conversely, cells did not respond to commercial S1P, either suggesting that S1P indeed requires a carrier molecule or that additional signaling factors are present in serum and triggering cell polarization. In this context, our experimental setup with a source of bioactive molecules and instant stimulus exposure represents a uniquely efficient working platform, granting further research to faithfully identify the bioactive molecules or complexes behind lymphocyte polarization. Meanwhile, with the available data we can readily conclude that under the same conditions in which CCL21 gradients triggered durable and long-range chemotaxis, serum factors, including bioactive S1P, did not. Instead, we hypothesize that serum factors provide a qualitative decision-making signal to trigger transmigration into cortical sinuses.

**Figure 7:**
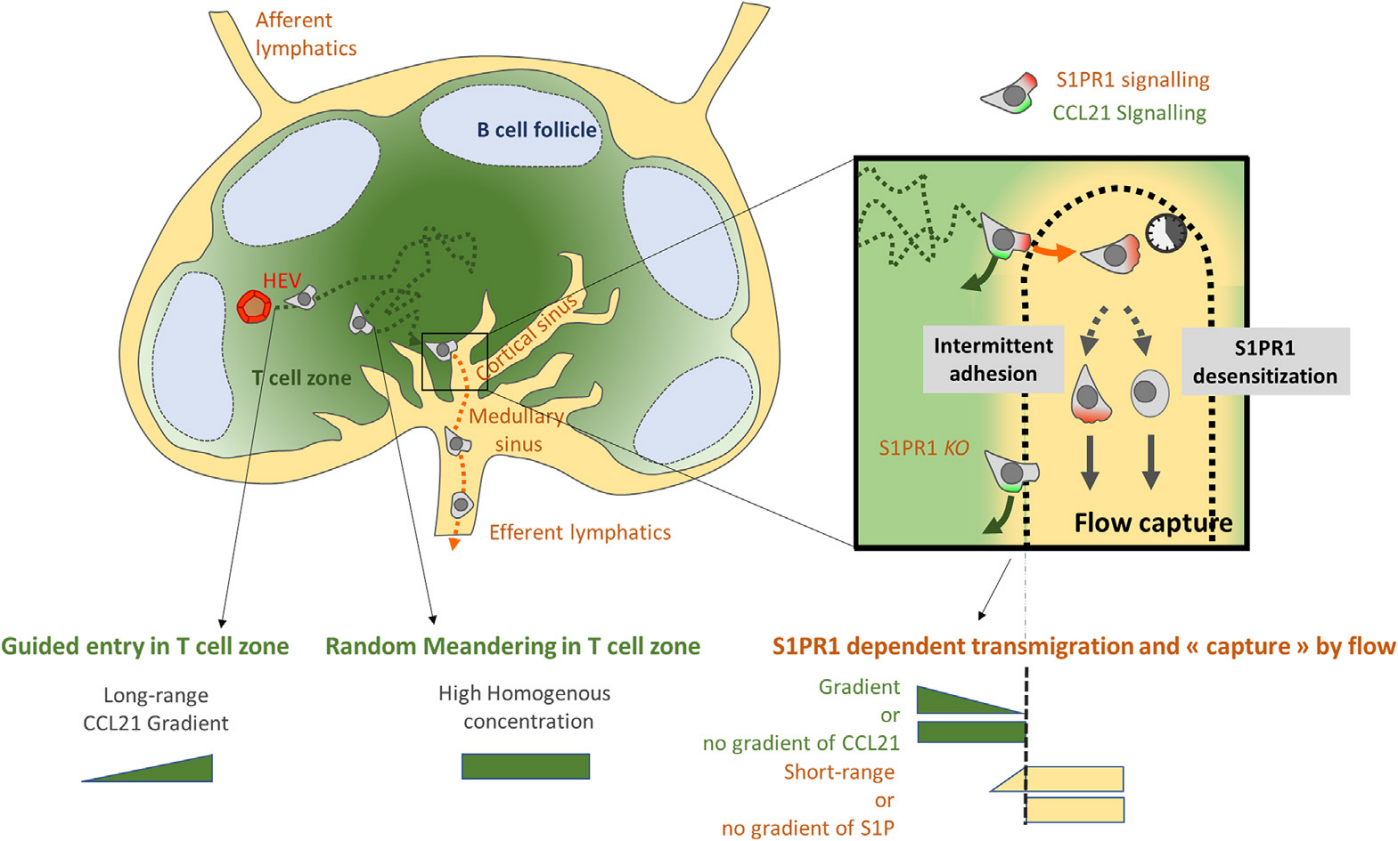
Illustration of a non-exhaustive minimal model for naive T lymphocyte trafficking in lymph nodes in the absence of T cell receptor activation The proposed scenario is based on the data showed here plus i) reported CCL21 gradients in lymph node peripheries,^7^^,^^8^^,^^9^ ii) random migration in the central parenchyma,^15^^,^^17^^,^^18^ iii) and random approach toward cortical sinuses, S1PR1-dependent transmigration and flow-capture of cells in the sinus.^16^ Naive T cells exit HEVs and migrate toward the central parenchyma guided by a long-range chemotaxis toward CCL21 (green), and then meander randomly in the T cell zone where CCL21 concentration is high and homogeneous. Naive T cell may then encounter a cortical sinus, either randomly (if there is no local S1PR gradient) or by short range guiding in the vicinity of a cortical sinus (if a S1P gradient exists there). Upon probing its lumen, S1PR1 sensing triggers the transmigration decision (which explains that S1P1 KO cells have deficient exit phenotypes). In the sinus, intermittent adhesion and S1PR1 internalization can independently trigger flow-capture and lymph node exit through efferent lymphatics.

Our data complement *in vivo* observations and leads to the following non-exhaustive model of naive T lymphocyte traffic (Figure 7). After accessing lymph nodes through HEVs or the subcapsular sinus floor, cells are gently attracted by long-range CCL19 and CCL21 gradients toward the central parenchyma, where they encounter higher and homogeneous concentrations of CCL21 that allow their random walk throughout the T cell zone. During this journey, they scan antigen-loaded DCs and can randomly encounter a cortical sinus. Upon probing its lumen, S1P signaling is needed to achieve successful transmigration, as S1PR1 KO cells are retained in lymph nodes.^16^ Since we did not observe any apparent serum-based inhibition of cell adhesion nor CCL21-triggered migration, the main role of S1P may be to force the transmigration step upon probing the cortical sinus lumen. A potential stromal gate function of LECs may also be at work, for instance by providing a scaffold for adhesion and transmigration or by delivering additional soluble or contact signals. Such notion is supported by the different S1P-independent basal speed observed for cells migrating in the medullary cords as compared to those in the parenchyma.^33^ Once in the lumen, intermittent adhesion and S1PR1 internalization (followed by cell depolarization) can both independently trigger the detachment of cells from LECs and their concomitant capture by the increasing flow of efferent lymph, which finally brings cells out of the lymph node and back into circulation.

Our data do not exclude the possibility of short-range S1P guidance (an order of magnitude lower than that of CCL21) attracting cells to cortical sinuses, but such guidance has been dismissed by *in vivo* experiments.^16^ We advocate that, considering the observed fast S1PR1 internalization, the absence of *in vivo* evidence supporting a S1P gradient or chemotaxis toward sinuses, and the observed lack of *in vitro* chemotaxis toward bioactive S1P, this molecule should not be considered chemotactic on naive T lymphocytes until faithful evidence arises. Instead, its ability to induce cell polarization *in vitro* and cell transmigration through endothelial portals *in vivo* should be further characterized in an experimental setup with biologically active S1P and instant stimulus sensing, as the one presented in Figure 6.

### Limitations of the study

Our results were obtained with naive T cells isolated from healthy donor blood samples and serum-starved to induce S1PR1 expression, thus mimicking the state of naive T cells in lymph nodes during their recirculation. However, in the light of other recent work,^33^ it would be interesting to examine the reactivity of other T cell sources. Furthermore, this study does not provide a molecular explanation as to why pure S1P is not bioactive in our assays, which molecule triggers phenotypic responses to bioactive serum, or why CCR7 and S1PR1 display such different down-regulation properties. To better understand the process of lymph node egress, it would be interesting to examine the potential internalization of CCR7 over long periods and the potential hierarchy between S1P and CCL21 signaling. These questions open up new avenues for future research.

## STAR★Methods

### Key resources table

**Table:** 

REAGENT or RESOURCE	SOURCE	IDENTIFIER
human IgG	LFB Biomedicaments	Tegeline
PE anti-human CD197 (CCR7)	Biolegend	clone G043H7; RRID: AB_10913813
PE/Cy7 anti-human CD62L	Biolegend	clone DREG-56; RRID: AB_830800
EF660 anti-human CD363 (S1PR1)	eBioscience	clone SW4GYPP; RRID: AB_2574208
EF660 IGG1K isotype control	eBioscience	Clone P3-6-2-8-1; RRID: AB_10597301
anti-human CD45RA antibody	Abcam	clone HI100; RRID: AB_10708880
Whole blood from healthy adult donors of group 0, drawn into EDTA tubes	Etablissements Francais du Sang, France	N/A
Ficoll gradient	Eurobio, Evry, France	N/A
Phosphate Buffer Saline (PBS)	Gibco, Carlsbad, CA	N/A
penicillin 100 U/ml	Gibco, Carlsbad, CA	N/A
streptomycin 100 μg/ml	Gibco, Carlsbad, CA	N/A
25 mM GlutaMax	Gibco, Carlsbad, CA	N/A
fetal calf serum	FCS, Lonza, Basel, Switzerland	N/A
1% paraformaldehyde (PFA)	PFA, Thermofisher	N/A
human ICAM-1	R&D Systems	N/A
1 μg/mL CCL21	Miltenyi biotech	N/A
fatty acid-free Bovine Serum Albumin	Sigma	N/A
mPEG-SVA (MW:5000 Da)	INTERCHIM	N/A
10 kDa Dextran FITC	Sigma	N/A
photoinitiator PLPP	Alveole, France	N/A
RBC lysis buffer	eBioscience	N/A
naïve CD4^+^ T Cell Isolation Kit II	Miltenyi	N/A
Ibidi μ-Slide uncoated IV 0.4	Ibidi GMBH, Martinsreid, Germany	N/A
pump system neMESYS 290N	Cetoni	N/A
Digital Micromirror Device Primo	Alveole	N/A

### Resource availability

#### Lead contact

Requests for additional information and resources should be directed to the lead contact, Olivier Theodoly (olivier.theodoly-lannes@cnrs.fr).

#### Materials availability

The recombinant proteins produced by the team of Daniel Legler may be available upon request from Daniel Legler. The microfluidic devices fabricated for this study and/or the design of the masks used for their fabrication may be available by contacting the lead contact.

### Experimental model and study participant details

#### Cells

Whole blood from healthy adult donors of group 0, drawn into EDTA tubes, was obtained from the “Établissement Français du Sang”. Peripheral Blood Mononuclear Cells (PBMC) were recovered from the interface of a Ficoll gradient (Eurobio, Evry, France) and washed with Phosphate Buffer Saline (PBS, Gibco). Naïve T lymphocytes were purified with the Miltenyi naïve CD4^+^ T Cell Isolation Kit II. After purification, cells were kept in RPMI 1640 medium supplemented with penicillin 100 U/ml (Gibco, Carlsbad, CA), streptomycin 100 μg/ml (Gibco, Carlsbad, CA), 25 mM GlutaMax (Gibco, Carlsbad, CA), with or without 10% fetal calf serum (FCS, Lonza, Basel, Switzerland) in a 37°C incubator with 5% CO_2_, until use. Human serum was prepared from the same donor, from blood coagulated in a dry tube. After 5 minutes centrifugation at 500 RCF the supernatant was taken, filtered through a 0.2 μm mesh and kept at 37°C until use.

### Method details

#### Flow cytometry

One hundred thousand cells per condition were fixed for 10 minutes with 1% paraformaldehyde (PFA, Thermofisher), then washed once with 4 mL of FACS buffer (2% FCS in PBS), resuspended in 100 μL and stained for 30 minutes at 4°C in the dark. They were finally washed with 4 mL FACS buffer and re-suspended in 0.5 mL to be analyzed in a LSR Fortessa X20 (BD Biosciences, Europe). For live staining, an equal number of purified cells or PBMCs were stained first on ice, then washed with FACS buffer and fixed with 1% PFA. For the blood sample, 200uL were first blocked with 1 mg human IgG (Tegeline, LFB Biomedicaments) for 15 minutes on ice, immediately after stained for 30 minutes, erythrocytes were then lysed with RBC lysis buffer (eBioscience), finally cells were washed with 12 mL PBS and fixed with 1% PFA. The antibodies used for staining were APC/Cyanine7 anti-human CD45RA (clone HI100, Biolegend), PE anti-human CD197 (CCR7, clone G043H7, Biolegend), PE/Cy7 anti-human CD62L (clone DREG-56, Biolegend), EF660 anti-human CD363 (S1PR1, clone SW4GYPP, eBioscience) and EF660 IGG1K isotype control (eBioscience).

#### Devices

Single channel devices consisted of Ibidi μ-Slide uncoated IV 0.4 (Ibidi GMBH, Martinsreid, Germany). Surfaces were coated overnight at 4°C, either with 10 μg/mL human ICAM-1 (R&D Systems), 1 μg/mL CCL21 (Miltenyi biotech) or a mixture of both, followed by blocking with 4% fatty acid-free Bovine Serum Albumin (BSA, Sigma) solution in PBS, for at least 15 min at room temperature. Devices were finally rinsed with PBS and then culture media. Flow experiments were performed in single channels connected to a pump system (neMESYS 290N, Cetoni). The gradient device was produced by soft photolithography and micromolding techniques. The mains channels were first fabricated by spin coating one layer of the negative photoresist SU-8 (MicroChem Newton, MA) (SU-8 3050, h=80 μm) on a 4-inch silicon wafer (Siltronix). The photoresist was exposed to UV light through the mask containing the gradient design. SU-8 developer solution (MicroChem, Newton, MA) was used to dissolve unexposed parts of the photoresist. The portions of channels to allow valve actuation were added by spin coating positive photoresist AZ-40XT at 500 rpm for 10s and 2800 rpm for 20s. Spin coating was followed by a 7min baking at 125°C, alignment and exposure to UV for 30s, a second bake for 1min and development in AZ326 MIF for 3.5min followed by a third bake for 7min. The molds for channels to actuate valve were produced on a separate wafer using SU-8 3050, h=40 μm. PDMS molding was performed by mixing the pre-polymer (Sylgard 184, Dow Corning) with the polymerization agent at 10:1 ratio for the devices and 12:1 ratio for the valve master molds. PDMS was directly poured on the device molds, followed by degassing in a vacuum bell and spin coated on the valve molds to obtain a thickness of 10 μm above the channels (actuator membrane). Molds where baked in a 65°C oven for at least 2 hours. After curing, only the gradient devices were unmolded and both PDMS surfaces (gradient devices and valve master with spin-coated PDMS) were treated in UV-Ozone for 30min, overlaid on top of each other and left overnight at 65°C to assure strong bonding. The next day the PDMS montages were removed from the valve molds and the inlets and outlets were punched with 1.2 or 2mm punchers (Harris Uni-Core), before being sealed on a glass slide via plasma activation (Harrick Plasma) for 15min and final 100°C baking for another 15min.^19^ 10 kDa Dextran FITC (Sigma) was used as a diffusion marker to analyze gradient dynamics. For CCL21 surface micropatterning, CCL21-S6^Dy549P1^ homogeneous substrates in single channels were UV-illuminated (λ = 375 nm, 300s exposition at 5V) through a Digital Micromirror Device (Primo; Alveole) in the presence of photoinitiator (PLPP, Alveole), to degrade the chemokine in a modulated way. Patterns of interest were designed on Matlab (The MathWorks). The fluorescent intensity was calibrated into number of molecules by imaging serial dilutions in 39 μm hight PDMS microchannels, passivated with PEG-SVA to limit adsorption, as described elsewhere.^59^ Substrates for cell capture were prepared on glass slides (Schott High performance coverslip 22x22#1.5H cleanroom cleaned) which were plasma activated (Harrick Plasma) during 30min and then incubated for 2 hours at 4°C with a 1% APTES 0.03% Acetic acid solution. Slides were then rinsed with Milli-Q water, dried and baked 15 min at 95°C. Open-wells (6x2mm) were finally created by sticking a 250μm-thick PDMS sticker on the slides. A solution of 23% mPEG-SVA (MW:5000 Da, INTERCHIM) 10mM NaHCO3 was then added to the wells, and substrates were incubated overnight at 4°C. The next day they were rinsed with Milli-Q water, and 10μl of PLPP solution (Alveole) was added. Arrays of capture spots, 3μm in radius, were illuminated with UV (λ=375nm for 180sec, ∼2488 mJ/mm^2^ dose) through a Digital Micromirror Device (Primo™, Alvéole). Wells were rinsed with PBS and incubated overnight with 50 μg/mL anti-human CD45RA antibody (Abcam ab212774, lot GR3365431-3), at 4°C.

#### Recombinant chemokine expression

pSUMO ΔCCL21 was cloned using the primers 5’-GGTGCTCGAGTCAGCCCTGGGCTGGTTTCTGTGGGGATGGTGTCTTG-3’ and 5’-CCCTCTAGAAATAATTTTGTTTAACTTTAAGAAGGAGATATACATATGG-3’ to amplify ΔCCL21 from pSUMO CCL21^26^ and introducing it into the pSUMO backbone using XbaI and XhoI cutting sites. The chemokines and dyes were produced as previously described.^47^ In brief, S6-tagged CCL21 and ΔCCL21 were each produced in E.coli BL21 DE3, refolded and using a multi step protocol affinity purified, with a final C18 reverse phase HPLC step. CoA-Dy549P1 was generated as described using DY-549P1-Maleimide (Dyomics GmbH, Germany) and CoA Li_3_ (Sigma-Aldrich, Switzerland). CoA-Dy549P1 was transferred to CCL21-S6 using the phosphopantetheinyl transferase Sfp and labelled chemokine purified using C18 reverse phase HPLC.

### Quantification and statistical analysis

#### Imaging and data analysis

Experiments were performed on an inverted Zeiss Z1 automated microscope (Carl Zeiss, Germany) equipped with a CoolSnap HQ CCD camera (Photometrics) and piloted by μManager 1.4. Plan-Apochromat 10x/0.3 and 20x/0.8 air objectives were used for bright-field imaging, while a 40x/1.3 oil one was used for Interference Reflection Microscopy (IRM). IRM images were first corrected by subtracting a background image, secondly the pixel values were inverted to convert dark signal into positive values, finally a rolling ball algorithm was applied to flatten the image. Cells were tracked using the FIJI plugin Trackmate,^60^ except for serum experiments where the Manual Tracking plugin was used. Tracks were exported and further analysis and plots were performed with MATLAB custom-made scripts (MATLAB software, The MathWorks, Natick, MA, USA). The unbiased colormap for the heatmaps was taken from ref.^61^ Gradient dynamics were analyzed with a custom-made FIJI macro, fluorescence intensity values were normalized with the equation:

$$\frac{I-I_{\min}}{I_{\max}-I_{\min}}\;x\;\left[C\right]$$

Where $I_{\min}$ is the average value recorded on the sink channel (background), $I_{\max}$ is the average value recorded on the source channel, and [C] is the chemokine concentration applied at the source. Ilastik^62^ was used for morphometric analysis, to create binary masks which were further processed with Fiji and the contour of each individual cell was analyzed with Matlab custom scripts versus time. The eccentricity of cell contours was used to determine whether cells were quiescent (eccentricity< 0.5) or polarized (eccentricity>0.5) and used to compute the percentage of polarized cells over time, as well as the duration of each polarization event, as illustrated in Figure 6.

Multiple comparison ANOVA tests were used to compare datasets. The number of analyzed cells, tested donors and resulting *p-*values are specified on each figure’s legend.

## Data Availability

• All data reported in this paper will be shared by the lead contact upon request. • This paper does not report original code. • Any additional information required to reanalyze the data reported in this paper is available from the lead contact upon request.
