# Supplementary material for: Naive T lymphocytes chemotax long distance to CCL21 but not to a source of bioactive S1P
Source: iScience. 2023 Aug 19;26(10):107695. doi: 10.1016/j.isci.2023.107695 (PMC10562802; doi:10.1016/j.isci.2023.107695)
Supplement: Document S1. Figures S1–S3 [file mmc1.pdf]

iScience, Volume *26*

Supplemental information

Naive T lymphocytes chemotax long distance to CCL21 but not to a source of bioactive S1P

Nicolas Garcia-Seyda, Solene Song, Valentine Seveau de Noray, Luc David-Broglio, Christoph Matti, Marc Artinger, Florian Dupuy, Martine Biarnes-Pelicot, Marie-Pierre Valignat, Daniel F. Legler, Marc Bajénoff, and Olivier Theodoly

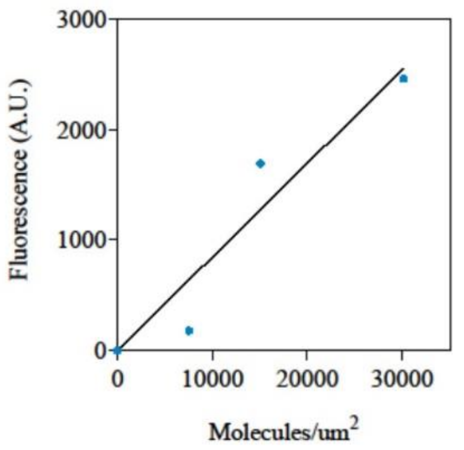

Supplementary Fig 1. Calibration of fluorescence signal for CCL21S6-Dy549P1 impressions, related to Figure 3.
Measurements of fluorescence intensity for CCL21S6-Dy549P1 solutions of known concentration in a channel of known thickness measured in the same optic conditions as in Fig 3E and 3F allows to build a calibration curve relating an experimental fluorescence intensity (A.U) to an effective surface concentration (molecules/μm^2).

A

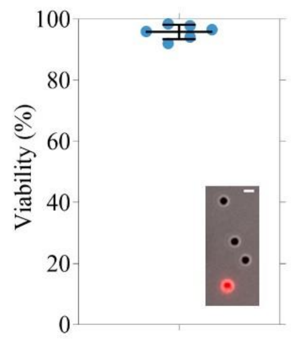

Supplementary Fig 2. **Viability and S1PR1 expression of naïve T cells after serum starving, related to Figure 4**.

(A) Viability of cells from 6 independently tested donors after >6hs incubation without serum. Inset presents the live and dead conditions, scored by propidium iodide uptake (red cell). Scalebar = 10µm (B) Left and center, purified naïve T lymphocytes from 12 independently tested donors were fixed, stained for S1PR1, and the percent of S1PR1 positive cells was scored. The gray histogram corresponds to the isotype control used to define the gates. Right, S1PR1 expression vs time, for cells purified from 1 donor and incubated in the absence of serum for the indicated timepoints, then fixed and stained for S1PR1.

B

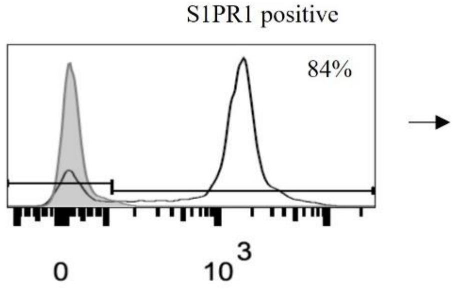

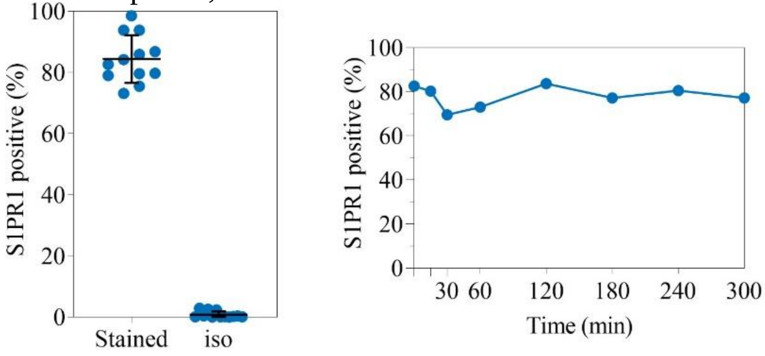

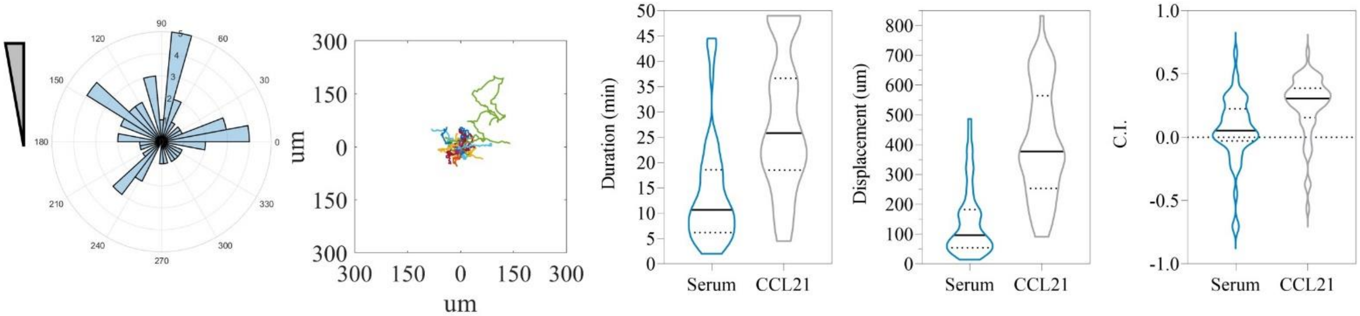

Supplementary Fig 3. **Comparaison of chemotaxis experiments S1P rich serum and CCL21 gradients, related to Figure .o** The data for S1P rich serum correspond to the tracking analysis for the cumulated 49 migrating cells (<8% of total cell population). From left to right, angle histogram, trajectories aligned in the origin, duration, displacement, and chemotactic index (C.I.) for those 49 tracks. As a comparison, same parameters for the 55 tracks shown in Fig 3B (CCL21 gradient, 1 representative donor) are plotted.
